# Public knowledge and attitudes towards bystander cardiopulmonary resuscitation (CPR) in Ghana, West Africa

**DOI:** 10.1186/s12245-020-00286-w

**Published:** 2020-06-10

**Authors:** Martina Anto-Ocrah, Nick Maxwell, Jeremy Cushman, Emmanuel Acheampong, Ruth-Sally Kodam, Christopher Homan, Timmy Li

**Affiliations:** 1 Department of Obstetrics and Gynecology, School of Medicine and Dentistry, University of Rochester, Rochester, NY USA; 2 Department of Neurology, School of Medicine and Dentistry, University of Rochester, Rochester, NY USA; 3 University of Rochester School of Medicine and Dentistry, Rochester, NY USA; 4 Department of Emergency Medicine, University of Rochester School of Medicine and Dentistry, Rochester, NY USA; 5 Department of Emergency Medicine, Komfo Anokye Teaching Hospital, Kumasi, Ghana; 6 Women and Children’s Health Advocacy Group-Ghana (https://wachagghana.org/), Accra, Ghana; 7 Department of Computer Sciences, Rochester Institute of Technology, Rochester, NY USA; 8 Department of Emergency Medicine, Donald and Barbara Zucker School of Medicine at Hofstra/Northwell, Manhasset, NY USA

**Keywords:** Social media, Cardiopulmonary resuscitation (CPR), Ambulance, Prehospital, Ghana

## Abstract

**Background and objectives:**

Early bystander cardiopulmonary resuscitation (CPR) is one of the most important predictors of out-of-hospital cardiac arrests (OHCA) survival. There is a dearth of literature on CPR engagement in countries such as Ghana, where cardiovascular events are increasingly prevalent. In this study, we sought to evaluate Ghanaians’ knowledge of and attitudes towards bystander CPR, in the context of the country’s nascent emergency medicine network.

**Methods:**

Capitalizing on the growing ubiquity and use of social media across the country, we used a novel social media sampling strategy for this study. We created, pre-tested, and distributed an online survey, using the two most utilized social media platforms in Ghana: WhatsApp and Facebook. An airtime data incentive of 5 US dollars, worth between 5 and 10 GB of cellular data based on mobile phone carrier, was provided as incentive. Inclusion criteria were (1) ≥ 18 years of age, (2) living in Ghana. Survey participants were encouraged to distribute the survey within their own networks to expand its reach. We stratified participants’ responses by healthcare affiliation, and further grouped healthcare workers into ambulance and non-ambulance personnel. We used chi-square (*χ2*)/Fisher’s Exact tests to compare differences in responses between the groups. Based on the question “have you ever heard of CPR?”, an alpha of 0.05 and a 95% confidence interval, we expected to have 80% power to detect a 15% difference in responses between lay and healthcare providers with an estimated sample size of 246 study participants.

**Results:**

The survey was launched on 8 July 2019 and closed approximately 51 h post-launch. With a 64% completion rate and 479 unique survey completions, the study was overpowered at 96% power, to detect differences in responses between the groups. There was geographic representation across all 10 historic regions of Ghana. Over half (57.8%, *n* = 277) of the respondents were non-medically affiliated, and 71.9% were women. Healthcare workers were more aware of CPR than lay respondents (96.5% vs 68.1%; *p* < 0.001). Eighty-five percent of respondents were aware that CPR involves chest compressions, and almost 70% indicated that “mouth to mouth” is a necessary component of CPR. Fewer than 10% were unwilling to administer CPR. Lack of skills (44.9%) and fear of causing harm (25.5%) were barriers noted by respondents for not administering CPR. Notably, a quarter of ambulance workers reported never having received CPR training. If they were to witness a collapse, 62.0% would call an ambulance, and 32.6% would hail a taxi.

**Conclusion:**

The majority of participants are willing to perform CPR. Contextualized training that emphasizes hands-only CPR and builds participants’ confidence may increase bystander willingness and engagement.

## Introduction

More than three million sudden cardiac deaths occur annually worldwide [1], with most occurring outside of a hospital, where survival is less than 8% [2]. Early bystander cardiopulmonary resuscitation (CPR) increases survival from out-of-hospital cardiac arrest (OHCA) by at least twofold [3, 4], whereas survival decreases by 7–10% each minute without CPR [5]. The rates of bystander CPR vary across countries, and ranges from 20 to 70% [2, 6–9]. These estimates however, are based on reports from resource-rich settings [1, 2, 6–13]. There is limited understanding of bystander CPR rates in low-to-middle income countries (LMICs), particularly those in the African Region, where cardiovascular-related morbidity and mortality are increasingly prevalent [14]. In a country like Ghana, where cardiovascular diseases (CVD) rank as one of the top two causes of mortality (second only to diarrheal diseases) [14, 15], life-saving measures such as CPR are critical in the OHCA setting to improve cardiovascular-related outcomes. Given CPR’s importance in OHCA, understanding the knowledge and attitudes of bystander CPR are critical to developing systems of care which have the potential to decrease the burden of cardiovascular conditions, while propelling the sustainable development goal of improving the health and well-being of all populations [16].

Across the African Region, prehospital systems are being developed to provide emergency care and transportation of patients [17–25]. In 2004, Ghana created the National Ambulance System (NAS), which provides free services to 81% of the country’s 27 million residents [17, 26]. In this study, we sought to evaluate Ghanaians’ knowledge of, attitudes towards, and noted barriers for performing bystander CPR. Aligning with the African Federation on Emergency Medicine (AFEM)’s consensus framework on approaching OHCA within African emergency care systems [20, 25], our intent is to identify contextually appropriate opportunities to improve bystander performance of CPR in Ghana, thereby improving OHCA survival.

## Methods

### Study setting and context

Ghana, approximately the size of the UK, has historically been divided into 10 regions, with 45% of the population spread over large, rural expanses of land [27, 28]. Cardiovascular diseases (CVD) rank as one of the top two causes of mortality [14, 15] in the country. In the capital city of Accra alone, CVDs have risen from the tenth to the leading cause of mortality over the last decade [29]. Social media is widely used in Ghana and its presence is growing rapidly. The West African country currently ranks in the top five for the largest social media growth in the last fiscal year [30, 31]. Internet penetration is an estimated 40% in Ghana, compared with 36% in India, 51% in South Africa, and 88% in North America [31]. Because of its ubiquity, we used a social media sampling strategy to recruit participants for this study.

### Online sampling and eligibility

We created an online survey (see Appendix) using REDCap (Research Electronic Data Capture, Vanderbilt University). Beginning with the social media networks of the Ghanaian authors (MAO, RSK and EA), we targeted Ghanaian Facebook and WhatsApp users ≥ 18 years of age to participate. Using a snowball sampling technique, the three authors distributed the survey to their social media networks (MAO: *n* = 12 WhatsApp contacts, RSK: *n* = 2000 Facebook followers and 500 WhatsApp group members, and EA: *n* = 300 WhatsApp contacts) and encouraged their contacts to forward it to their social networks. Upon clicking the survey link, participants were brought to a page that detailed the research purpose, risk/benefits of participating, incentives for participating, and consenting process. Once consented, participants were prompted to complete the survey.

An airtime data incentive of 5 US dollars, worth between 5 and 10 GB of cellular data based on mobile phone carrier, was provided to those who completed the survey and provided their phone numbers. This form of incentives allows participants to defray data-related expenses [32]. Participants were encouraged to share the link to increase the survey’s distribution and to encourage further participation. The study was approved by the University of Rochester’s Institutional Review Board.

### Survey questionnaire

The survey (Appendix) collected demographic information and asked CPR-specific questions derived from prior literature [1, 8, 10, 13]. All survey questions were reviewed for relevance and context by the research team members. CPR content was reviewed by NM, JC, EA, and TL; while MAO, EA, and RSK reviewed the survey for cultural appropriateness. The survey was iteratively pilot-tested to ensure that it was conducive to the Ghanaian mobile telephone platform.

To assess CPR knowledge, participants were asked to define CPR, when CPR should be administered, whether CPR requires both chest compressions and mouth-to-mouth ventilation, and to indicate whether they received any CPR training. Attitude-based questions evaluated participants’ feelings about administering CPR to various members of their community, and the barriers. They were also presented with a scenario [8] and asked to identify the actions they would take.

### Analyses

Descriptive statistics were used to describe the study sample. The sample was stratified into two self-identified groups: (1) those who worked in the medical field, and (2) those who did not work in the medical field. We further grouped medically affiliated respondents by ambulance experience, based on yes/no responses to the question “Do you work with ambulances?” Differences between the groups were assessed using chi-square or Fisher’s exact tests where appropriate. Age was the only continuous variable and difference in age was assessed using the Wilcoxon rank sum test, due to its non-parametric attributes. We used *p* < 0.05 to determine statistical significance for all analyses.

We operationalized CPR as an “emergency procedure in which chest compressions are administered to provide artificial circulation, which may or may not include artificial ventilations.” We used a coding scheme of “accurate”, “close”, and “inaccurate” to categorize participants’ responses to the question “In your own words, what is CPR or cardiopulmonary resuscitation?” and “Why would someone need CPR or cardiopulmonary resuscitation?” JC and NM independently coded the responses to these two questions, and EA resolved any discrepancies, keeping in mind the contextual nature of the study, and the vast heterogeneity of the study participants.

### Sample size determination

We estimated the prevalence of bystander CPR in the OHCA setting among participants to be at least 20%, aligning with low threshold of prevalence estimates cited across the literature [6, 7]. Based on this 20% prevalence estimate, an alpha of 0.05 and a 95% confidence interval, an estimated sample size of 246 was required [33]. We over-estimated our sample size by 50% to ensure adequate reach and representation of participants across the country.

## Results

**Fig. 1:**
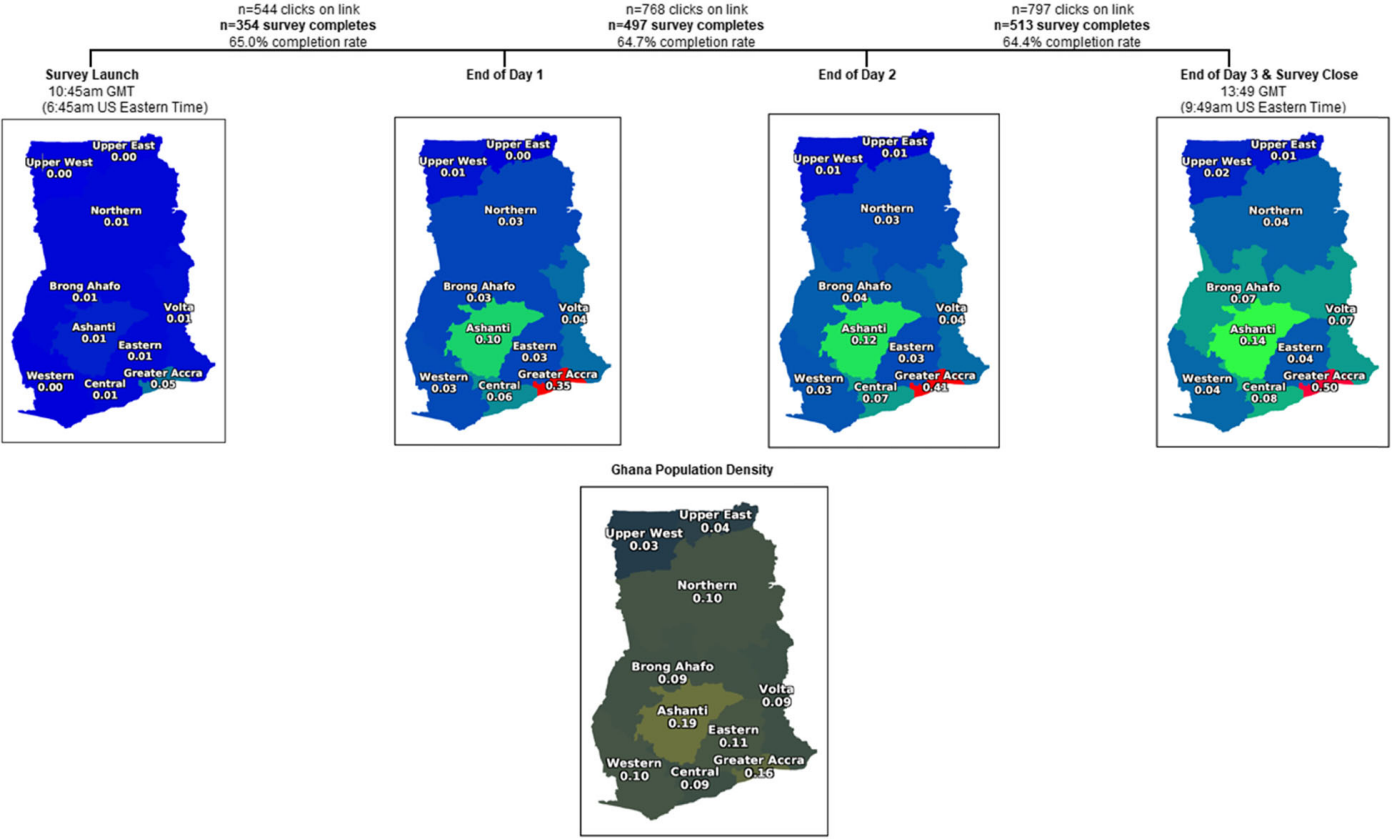
Distribution and reach of bystander CPR survey in Ghana, West Africa

The survey was launched on 8 July 2019 and closed approximately 51 h post-launch (Fig. 1). There were 797 clicks on the link and 513 eligible individuals completed the survey. Half of the study participants hailed from Greater Accra, despite the region being home to only 16% of Ghana’s population [27, 34]. Relative to population estimates, the eastern (0.04 survey density vs 0.11 population density), northern (0.04 survey density vs 0.10 population density), and western (0.04 survey density vs 0.10 population density) regions were particularly under-represented.

Of the 513 completed surveys, 29 were excluded due to having duplicate phone numbers and 5 were excluded due to not answering the question “Do you work in the medical field?” With a final sample size of 479 participants, the study was overpowered at 96%. As shown in Table 1, 57.8% (*n* = 277) of the 479 participants were not medically affiliated and 42.2% (*n* = 202) identified as healthcare workers. Median age of the sample was 30 years (interquartile range 27, 34), 71.9% were female, 50.7% were married/co-habitating, and 86.6% had at least a university education. Though age differences between the medical and non-medical professionals were not statistically significant (*p* = 0.139), there were significant differences in educational attainment between the groups. A greater proportion of the non-medical group had lower educational attainment compared with medical professionals (11.0% vs 4.04%, *p* = 0.004). Among healthcare workers, only 38.4% worked with ambulances. Greater Accra and Ashanti Region, the two most populous Ghanaian Regions (Fig. 1), had the greatest representation of healthcare workers.

**Table 1:** Demographic characteristics^b^

Characteristic	Total sample	Work in medical field	Do not work in medical field	*p* value*
Age, years	***n*= 479**	***n*= 202**	***n*= 277**	0.139
Median, (25th, 75th percentile)	30 (27, 34)	30 (26, 33)	30 (27, 36)	
Mean (±SD)	31 (±6.8)	33 (±5.2)	32 (±7.7)	
Range	19–74	19–60	20–74	
Sex	***n*= 476**	***n*= 201**	***n*= 275**	0.362
Female, *n* (%)	342 (71.9%)	140 (69.7%)	202 (73.5%)	
Male, n (%)	134 (28.2%)	61 (30.4%)	73 (26.6%)	
Marital status	***n*= 475**	***n*= 202**	***n*= 273**	0.471
Married/cohabitating, *n* (%)	241 (50.7%)	105 (52.0%)	136 (49.8%)	
Single/widowed, *n* (%)	228 (48.0%)	96 (47.5%)	132 (48.4%)	
Divorced/separated, *n* (%)	6 (1.3%)	1 (0.5%)	5 (1.8%)	
Highest level of education	***n*= 471**	***n*= 198**	***n*= 273**	0.004
No schooling, *n* (%)	1 (0.2%)	0 (0.0%)	1 (0.4%)	
Junior/senior high/technical school, *n* (%)	38 (8.1%)	8 (4.0%)	30 (11.0%)	
University/postgraduate, *n* (%)	408 (86.6%)	174 (87.9%)	234 (85.7%)	
Other, n (%)	24 (5.1%)	16 (8.1%)	8 (2.9%)	
Work with ambulances^a^	***n*= 190**	***n*= 190**		N/A
Yes, *n* (%)	73 (38.4%)	73 (38.4%)	–	
No, *n* (%)	117 (61.6%)	117 (61.6%)	–	
Region of residence in Ghana	***n*= 479**	***n*= 202**	***n*= 277**	< .001
Ashanti Region, *n* (%)	70 (14.6%)	41 (20.3%)	29 (10.5%)	
Brong Ahafo Region, *n* (%)	30 (6.3%)	19 (9.4%)	11 (4.0%)	
Central Region, *n* (%)	38 (7.9%)	17 (8.4%)	21 (7.6%)	
Eastern Region, *n* (%)	18 (3.8%)	10 (5.0%)	8 (2.9%)	
Greater Accra Region, *n* (%)	240 (50.%)	72 (35.6%)	168 (60.7%)	
Northern Region, *n* (%)	20 (4.2%)	11 (5.5%)	9 (3.3%)	
Upper East Region, *n* (%)	5 (1.0%)	3 (1.5%)	2 (0.7%)	
Upper West Region, *n* (%)	7 (1.5%)	4 (2.0%)	3 (1.1%)	
Volta Region, *n* (%)	30 (6.3%)	18 (8.9%)	12 (4.3%)	
Western Region, *n* (%)	21 (4.4%)	7 (3.5%)	14 (5.1%)	

^*^p values derived from Wilcoxon rank sum test for age and chi-square tests or Fisher’s exact test for all other variables

^a^Only asked of those who work in medical field

^b^Item totals may not sum to total sample size (*n* = 479) due to missing data

As shown on Table 2, 8 out of 10 participants had heard of CPR, with greater familiarity amongst healthcare workers (95.5% vs 68.1%; *p* < 0.001). Non-healthcare workers more frequently indicated they were “not sure” when asked about CPR-specific procedures, including chest compressions (22.3% vs 2.3%; *p* < 0.001) and necessity of “mouth-to-mouth” resuscitation for CPR (30.6% vs 7.0%; *p* < 0.001). Over 90% of the non-healthcare workers reported they had never received CPR training and 25.4% of ambulance workers also reported that they had never received CPR training (*p* = 0.006). Over 90% of participants were interested in receiving CPR training; though across all groups, a non-negligible proportion (3.0% medical, 12.5% non-medical (*p* < 0.001), 2.7% ambulance, 3.4% non-ambulance (*p* = 0.712)) were “not sure” of the need to be CPR trained.

**Table 2:** Cardiopulmonary resuscitation knowledge and training^a^

	Total study sample	Work in medical field	Do not work in medical field	*p* value*	Total responding to “Do you work with Ambulances?”	Work with ambulance	Do not work with ambulances	*p* value*
Have you ever heard of CPR or cardiopulmonary resuscitation?								
	***n*= 471**	***n*= 198**	***n*= 273**		***n*= 186**	***n*= 70**	***n*= 116**	
Yes, *n* (%)	377 (80.0%)	191 (96.5%)	186 (68.1%)	< .001	179 (96.2%)	68 (97.1%)	111 (95.7%)	0.713
No, *n* (%)	94 (20.0%)	7 (3.5%)	87 (31.9%)		7 (3.8%)	2 (2.9%)	5 (4.3%)	
Does CPR include chest compressions?								
	***n*= 419**	***n*= 177**	***n*= 242**		***n*= 170**	***n*= 62**	***n*= 107**	
Yes, *n* (%)	358 (85.4%)	172 (97.2%)	186 (76.9%)	< .001	165 (97.1%)	61 (96.8%)	104 (97.2%)	0.767
No, *n* (%)	3 (0.7%)	1 (0.6%)	2 (0.8%)		1 (0.6%)	0 (0.0%)	1 (0.9%)	
Not sure, *n* (%)	58 (13.8%)	4 (2.3%)	54 (22.3%)		4 (2.4%)	2 (3.2%)	2 (1.9%)	
Does CPR require “mouth to mouth”?								
	***n*= 465**	***n*= 200**	***n*= 265**		***n*= 189**	***n*= 72**	***n*= 117**	
Yes, *n* (%)	322 (69.3%)	160 (80.0%)	162 (61.1%)	< .001	150 (79.4%)	54 (75.0%)	96 (82.1%)	0.122
No, *n* (%)	48 (10.3%)	26 (13.0%)	22 (8.3%)		25 (13.2%)	14 (19.4%)	11 (9.4%)	
Not sure, *n* (%)	95 (20.4%)	14 (7.0%)	81 (30.6%)		14 (7.4%)	4 (5.6%)	10 (8.6%)	
Have you ever taken a CPR or cardiopulmonary resuscitation training class?								
	***n*= 470**	***n*= 199**	***n*= 271**		***n*= 188**	***n*= 71**	***n*= 117**	
Yes, *n* (%)	146 (31.1%)	124 (62.3%)	22 (8.1%)	< .001	117 (62.2%)	53 (74.7%)	64 (54.7%)	0.006
No, *n* (%)	324 (68.9%)	75 (37.7%)	249 (91.9%)		71 (37.8%)	18 (25.4%)	53 (45.3%)	
When was the last time you attended a CPR training class?								
	***n*= 143**	***n*= 121**	***n*= 22**		***n*= 114**	***n*= 51**	***n*= 63**	
1 year ago, *n* (%)	49 (34.3%)	47 (38.8%)	2 (9.1%)	< .001	44 (38.6%)	23 (45.1%)	21 (33.3%)	0.347
2 year ago, *n* (%)	24 (16.8%)	19 (15.7%)	5 (22.7%)		18 (15.8%)	9 (17.7%)	9 (14.3%)	
3 year ago, *n* (%)	29 (20.3%)	28 (23.1%)	1 (4.6%)		27 (23.7%)	10 (19.6%)	17 (27.0%)	
4 year ago, *n* (%)	15 (10.5%)	13 (10.7%)	2 (9.1%)		12 (10.5%)	6 (11.8%)	6 (9.5%)	
5 year ago, *n* (%)	26 (18.2%)	14 (11.6%)	12 (54.6%)		13 (11.4%)	3 (5.9%)	10 (15.9%)	
Would you be interested in receiving training in CPR or cardiopulmonary resuscitation?								
	***n*= 472**	***n*= 200**	***n*= 272**		***n*= 189**	***n*= 73**	***n*= 116**	
Yes, *n* (%)	426 (90.3%)	192 (96.0%)	234 (86.0%)	0.001	181 (95.8%)	71 (97.3%)	110 (94.8%)	0.718
No, *n* (%)	6 (1.3%)	2 (1.0%)	4 (1.5%)		2 (1.1%)	0 (0.0%)	2 (1.7%)	
Not sure, *n* (%)	40 (8.5%)	6 (3.0%)	34 (12.5%)		6 (3.2%)	2 (2.7%)	4 (3.5%)	

^*^p values derived from chi-square tests or Fisher’s exact test

^a^Item totals may not sum to total sample size (*n* = 479) due to missing data

Study participants were more willing to administer CPR to a spouse/partner (81.0%), a relative (80.6%), and a child (77.5%) than all other members of their community (Table 3). Few were unwilling to administer CPR (6.9% medical, 8.7% non-medical (*p* = 0.488); 5.5% ambulance, 6.0% non-ambulance (*p* = 1.000)). When asked about reasons why they would not administer CPR, many non-healthcare workers indicated they lack the skillset to administer CPR (61.4% vs 22.3%; *p* < 0.001), and about a third indicated they fear harming the patient (34.7% vs 12.9%; *p* < 0.001). A higher proportion of medical than non-medically affiliated respondents indicated fear of giving mouth-to-mouth resuscitation would prevent them from administering CPR (10.9% vs 5.4; *p* = 0.027). A significant proportion of non-ambulance versus ambulance providers indicated they would not administer CPR for fear of catching a disease (22.2% vs 9.6%; *p* = 0.025).

**Table 3:** Attitudes and willingness to perform cardiopulmonary resuscitation

	Total sample (*n* = 479)	Work in medical field (*n* = 202)	Do not work in medical field (*n* = 277)	*p* value*	Total responding to “Do you work with Ambulances?” (*n* = 190)	Work with ambulances (*n* = 73)	Do not work with ambulances (*n* = 117)	*p* value*
I would give CPR to someone if they are...^a^								
A child, *n* (%)	371 (77.5%)	171 (84.7%)	200 (72.2%)	0.001	161 (84.7%)	63 (86.3%)	98 (83.8%)	0.636
My spouse/partner, *n* (%)	388 (81.0%)	175 (86.6%)	213 (76.9%)	0.007	165 (86.8%)	65 (89.0%)	100 (85.5%)	0.479
A stranger, *n* (%)	282 (58.9%)	155 (76.7%)	127 (45.9%)	< .001	146 (76.8%)	62 (84.9%)	84 (71.8%)	0.037
My relative *n* (%)	386 (80.6%)	171 (84.7%)	215 (77.6%)	0.055	161 (84.7%)	64 (87.7%)	97 (82.9%)	0.374
My neighbor *n* (%)	333 (69.5%)	168 (83.2%)	165 (59.6%)	< .001	159 (83.7%)	64 (87.7%)	95 (81.2%)	0.240
Other, *n* (%)	59 (12.3%)	40 (19.8%)	19 (6.9%)	< .001	36 (19.0%)	18 (24.7%)	18 (15.4%)	0.113
No one, *n* (%)	38 (7.9%)	14 (6.9%)	24 (8.7%)	0.488	11 (5.8%)	4 (5.5%)	7 (6.0%)	1.000
Why would you not want to give someone CPR?^a^								
I fear I may catch a disease, *n* (%)	83 (17.3%)	34 (16.8%)	49 (17.7%)	0.807	33 (17.4%)	7 (9.6%)	26 (22.2%)	0.025
I do not have the skills to give CPR, *n* (%)	215 (44.9%)	45 (22.3%)	170 (61.4%)	< .001	40 (21.1%)	11 (15.1%)	29 (24.8%)	0.110
I may not know the person, *n* (%)	37 (7.7%)	13 (6.4%)	24 (8.7%)	0.367	13 (6.8%)	5 (6.9%)	8 (6.8%)	1.000
I do not have the confidence to give CPR, *n* (%)	70 (14.6%)	25 (12.4%)	45 (16.3%)	0.237	21 (11.1%)	8 (11.0%)	13 (11.1%)	0.974
I may harm the person or make things worse, *n* (%)	122 (25.5%)	26 (12.9%)	96 (34.7%)	< .001	22 (11.6%)	10 (13.7%)	12 (10.3%)	0.471
I do not want to give mouth to mouth, n (%)	37 (7.7%)	22 (11.0%)	15 (5.4%)	0.027	21 (11.1%)	6 (8.2%)	15 (12.8%)	0.325
I am afraid of getting sued, *n* (%)	45 (9.4%)	23 (11.4%)	22 (7.9%)	0.202	22 (11.6%)	6 (8.2%)	16 (13.7%)	0.253
Other reasons, *n* (%)	9 (1.9%)	6 (3.0%)	3 (1.1%)	0.133	5 (2.6%)	2 (2.7%)	3 (2.6%)	1.000
I will always give CPR, *n* (%)	165 (34.5%)	120 (59.4%)	45 (16.3%)	< .001	112 (59.0%)	47 (64.4%)	65 (55.6%)	0.229
You are at home in the evening. Suddenly you hear a loud noise in the kitchen. You burst in and find a close family member lying lifeless on the floor. He or she is not breathing. You are alone in the house. What will you do?^a^								
Call for ambulance, *n* (%)	297 (62.0%)	145 (71.8%)	152 (54.9%)	< 0.001	137 (72.1%)	55 (75.3%)	82 (70.1%)	0.432
Call for taxi, *n* (%)	156 (32.6%)	58 (28.7%)	98 (35.4%)	0.124	54 (28.4%)	18 (24.7%)	36 (30.8%)	0.364
Call for others to help, *n* (%)	295 (61.6%)	142 (70.3%)	153 (55.2%)	< 0.001	134 (70.5%)	54 (74.0%)	80 (68.4%)	0.411
Give them mouth to mouth, *n* (%)	243 (50.7%)	132 (65.4%)	111 (40.1%)	< 0.001	124 (65.3%)	49 (67.1%)	75 (64.1%)	0.671
Give them chest compressions, *n* (%)	331 (69.1%)	169 (83.7%)	162 (58.5%)	< 0.001	159 (83.7%)	66 (90.4%)	93 (79.5%)	0.048
Do something else, *n* (%)	35 (7.3%)	25 (12.4%)	10 (3.6%)	< 0.001	23 (12.1%)	12 (16.4%)	11 (9.4%)	0.148
I’m not sure what I will do, *n* (%)	24 (5.0%)	2 (1.0%)	22 (7.9%)	< 0.001	1 (0.5%)	1 (1.4%)	0 (0.0%)	0.384

*p* values derived from chi-square tests or Fisher’s exact test

^a^Response options are not mutually exclusive

When presented with a scenario necessitating CPR (Table 3), 69.1% of respondents indicated that they would perform chest compressions, 62.0% would call an ambulance, 50.7% would administer mouth-to-mouth resuscitation, and 32.6% would hail a taxi to transport the patient to the nearest hospital.

When asked to define CPR, 49.9% of respondents were able to provide a “close” definition (Table 4 and Data-in-Brief) respondents either misunderstood the indication for CPR (43.3%), the physiological basis of the procedure (35.6%), or had a misunderstanding of CPR procedures (21.6%). The question “Why would someone need CPR?” showed significant differences between medical and non-medical respondents, with the latter having a significantly higher proportion of inaccurate responses (3.8% vs 19.7%; *p* < 0.001).

**Table 4:** Accuracy of CPR definitions and indication, as provided by study participants^a^

	Total study sample	Work in medical field	Do not work in medical field	*p* value*	Total responding to “Do you work with Ambulances?”	Work with ambulances	Do not work with ambulances	*p* value*
“In your own words what is CPR or cardiopulmonary resuscitation?”								
	***n*= 417**	***n*= 185**	***n*= 232**		***n*= 175**	***n*= 66**	***n*= 109**	
CPR definition accurate, n (%)	162 (38.9%)	93 (50.3%)	69 (29.7%)	< 0.001	87 (49.7%)	38 (57.6%)	49 (45.0%)	0.154
Close definition of CPR n (%)	208 (49.9%)	88 (47.6%)	120 (51.7%)		84 (48.0%)	26 (39.4%)	58 (53.2%)	
CPR definition inaccurate, n (%)	47 (11.3%)	4 (2.2%)	43 (18.5%)		4 (2.3%)	2 (3.0%)	2 (1.8%)	
“Why would someone need CPR or cardiopulmonary resuscitation”?								
	***n*= 409**	***n*= 186**	***n*= 223**		***n*= 176**	***n*= 66**	***n*= 110**	
Accurate indication for CPR, *n* (%)	222 (54.3%)	128 (68.8%)	94 (42.2%)	< 0.001	120 (68.2%)	54 (81.8%)	66 (60.0%)	0.005
Close indication for CPR, *n* (%)	136 (33.3%)	51 (27.4%)	85 (38.1%)		49 (27.8%)	10 (15.2%)	39 (35.5%)	
Inaccurate indication for CPR, *n* (%)	51 (12.5%)	7 (3.8%)	44 (19.7%)		7 (4.0%)	2 (3.0%)	5 (4.6%)	

^*^p values derived from chi-square tests or Fisher’s exact test

^a^Item totals may not sum to total sample size (*n* = 479) due to missing data

## Discussion

Since the rate of OHCA and the capacity of the NAS are both rising rapidly in Ghana, engaging the public as CPR-trained responders could drastically decrease morbidity and mortality. In this study, we used a novel snowball sampling technique by leveraging social media to share an online survey and remove barriers to participation by offering a small data bundle as incentive. The rapidity of survey completion and the distribution of the survey throughout all regions of Ghana offer great promise to engage communities of interest to improve public health.

CPR knowledge varied among study participants. Over 80% of respondents had heard of the procedure and bystander willingness to engage in CPR was also high. Similar to prior literature from resource-rich settings [2, 7, 8, 10], over 90% of Ghanaians in this study were willing to administer CPR to anyone who needed it; and only 7.9% indicated they would not administer the procedure when necessary. However, a non-negligible proportion of both medical and non-medically affiliated respondents were unsure of the appropriate procedures for CPR administration. Over half of respondents considered mouth-to-mouth resuscitation a necessary component of CPR. This finding echoes that of surveyed Taiwanese [10] and Scottish [13] residents, who also identify mouth-to-mouth resuscitation as a barrier to performing CPR. In a 2019 study, Huang et al. [10] report that approximately 60% of surveyed Taiwanese residents would perform CPR on strangers if they did not need to perform mouth-to-mouth resuscitation, and 94% of those surveyed would prefer compressions-only CPR on unknown people if they had the skills. Importantly, the American Heart Association recommends compressions or hands-only CPR without mouth-to-mouth breaths [9] for bystanders. However, similar to surveyed populations in “high-resource” settings, this knowledge may not be common among Ghanaians. Thus while capitalizing on the enthusiasm for bystander engagement, training efforts in Ghana should continuously emphasize the importance of hands-only CPR without mouth-to-mouth resuscitation to overcome this barrier. This may also allay participants’ fear of “catching a disease,” which was a significant differentiator amongst healthcare workers who work with ambulances and those who do not. Knowing that mouth-to-mouth resuscitation is not required for CPR may improve training initiatives and bystander engagement.

One in four ambulance providers who participated in our study indicated they have never received CPR training. This is in stark contrast to the level of training available to individuals in most other countries represented in the literature. In Norway, the United States, Japan, and China, CPR training is offered not only to healthcare providers, but even to middle and high school students [1, 8]. Over 89% of secondary/high school students in Norway have access to CPR training [8], while more than half of students in the USA learn CPR in combination with automated external defibrillator use [1]. In Japan, over 30% of surveyed respondents indicated that they have learned CPR more than twice [1], and 27% of Chinese students have access to CPR training [1]. Though CPR training is offered to ambulance providers in Ghana [35], access to such training may be limited and these results suggest an opportunity for mandating such training. CPR is one of the most rudimentary and impactful interventions that an EMS provider can administer in the OHCA setting, especially in Ghana, where cardiovascular diseases rank high [14, 15, 29]. With 97% of ambulance providers indicating that they would be interested in receiving the training, additional resources are necessary to ensure that these providers are well equipped to provide the care patients need.

Repeatedly, we also observed that respondents often equated a “heart attack” with “cardiac arrest.” This raises the concern that there may be a lack of understanding surrounding the signs and symptoms of a heart attack (myocardial infarction). If so, this suggests an important opportunity to reduce the overall burden of sudden cardiac death by developing systems of care surrounding coronary syndromes within the Ghanaian context.

Another interesting finding that emerged from our study was the use of taxis for emergency transport, rather than ambulances. Previously published literature on ambulance knowledge and use in Ghana suggests Ghanaians prefer to use taxis as modes of emergency transport over ambulances [26, 36]. In our study, however, a great majority of respondents indicated that they would call an ambulance rather than a taxi if they were to witness a sudden collapse. Six in 10 study participants indicated they would call an ambulance, compared to 32.6% who showed a preference for taxi services. This includes over half of the non-medical survey respondents. Since the NAS was created 15 years ago, the Ministry of Health has made substantial efforts to raise awareness of emergency medical services and its saving capabilities [35]. These efforts may have increased acceptance of the NAS, but we are unable to substantiate these findings from our results as our study did not directly evaluate the public’s view of the NAS. Future research should evaluate how popular opinion of ambulances and other forms of prehospital care (automated external defibrillators, for example) evolve over time. Longitudinal studies evaluating such outcomes would be particularly helpful by providing guidance for allocating resources for various healthcare interventions, particularly for the nascent emergency care systems evolving across Africa.

To our knowledge, this is the first study to evaluate bystander CPR knowledge and attitude among Ghanaians. Our use of social media sampling enhanced not only the study’s novelty, but also touches on the relevance of mobile phone technology and the centrality of social media use among Ghanaians and possibly, other African populations. This approach allowed us to complete data collection quickly, while ensuring appropriate representation of respondents from each of the country’s regions.

There are several limitations of this study to acknowledge. First is the bias in sampling. Even though cell phones are ubiquitous across Ghana, access to such technology and/or social media is not. Getting online is often cost-prohibitive for many, as mobile data is expensive [32]. Our sampling approach excluded those who do not have cell phones, mobile data, or access to social media. This limitation is emphasized by the over-representation of participants from Greater Accra, one of the most urban regions of Ghana where residents tend to be more affluent and have more access to technology compared to residents in rural communities [34, 37]. Therefore, our study is limited in its generalizability to a technologically enabled-and therefore economically advantaged-population. Even though it may seem anachronistic given the ongoing social media boom, studies that use “traditional” recruiting strategies should be conducted to substantiate our findings and ensure the various views of Ghanaians across all socio-economic strata are represented. Findings from such studies can be used in conjunction with ours to develop targeted CPR training programs to engage Ghanaians. In addition to the geographic limitations imposed by our sampling technique, there is a potential for age bias as well. The age differences between the medical and non-medical professionals were not statistically significant, and our study participants skewed “younger,” with an average age of 31(± 6.8) and an age range of 19–74. “Typical” CVD outcomes affect “older” individuals who may have “older” spouses and family members. However, epidemiological data show global discrepancies in CVD-related outcomes. More than 50% of CVD-related deaths in the African Region occur among individuals between the ages of 30–69 years of age, which is 10 years or more below the equivalent group in non-African settings [15]. Therefore even though our study population may appear younger, they in fact approximate the age range of those most likely to be affected by CVD in this context, as well as those most likely to be in need of, and to initiate bystander CPR interventions. This finding highlights even more the urgency, relevance, and importance of our study and the need for interventions to abate these profound outcomes in Ghana.

Second, there are interesting biases due to snowball sampling. The phenomenon of homophily (that people tend to associate with those similar to themselves) probably led to such a large representation of medical professionals responding to the survey, as well as the large proportion of female respondents. It may also mean that non-medical respondents, due to their proximity to medical professionals, know more about CPR than the average Ghanaian. Thus, research representative of a more lay and gender-balanced population are needed to verify the findings.

Third, we only included those ≥ 18 years of age. The extent to which Ghanaians < 18 years are aware of and willing to engage in CPR was not evaluated in our study. Given that Ghanaians as young as 30 are succumbing to cardiovascular diseases [15, 29], “younger” citizens may be more likely to witness OHCA and feel compelled to administer CPR. Thus empowering those < 18 years may increase CPR-related outcomes in the Ghanaian context. Studies that assess the knowledge, attitudes, and willingness to engage in CPR by those < 18 are needed to guide such preventative efforts.

## Conclusion

Ghanaians who participated in our study are willing and interested in initiating bystander CPR. CPR training is desired by both medical professionals and lay persons and should stress the importance of hands-only CPR rather than mouth-to-mouth resuscitation to improve bystander engagement, confidence, and allay fears of disease transmission. The use of social media to engage interested research participants to evaluate their willingness to perform lifesaving procedures, albeit with noted limitations, is both a novel and effective means of sampling a population.

## Data Availability

Survey included as Appendix in manuscript. The datasets during and/or analyzed during the current study are available from the corresponding author on reasonable request.

## Appendix

### Appendix-survey questionnaire

**Demographics**

Q1.Are you

under 18 years of age (exclude)

18 years of age or older

Q2.Do you live in Ghana?

Yes

No (exclude)

Q3.Please what is your age in years (e.g 21, 32, 51 etc) __________________________________

Q4. Which region of Ghana do you live in?

Ahafo Region

Ashanti Region

Bono East Region

Brong Ahafo Region

Central Region

Eastern Region

Greater Accra Region

North East Region

Northern Region

Oti Region

Savannah Region

Upper East Region

Upper West Region

Volta Region

Western Region

Western North Region

Q5. Are you..

Female

Male

Q6. Are you currently

Single

Married

Divorced/Separated

Widowed

Other (specify)

Q7.What is your highest level of education?

Primary School (US Grades 1-6)

Junior/Senior High School/Technical School (USGrades 7-12)

University/ Postgraduate

I have had no schooling

Other (specify)

Q8.Do you work in the medical field (doctor/nurse/midwife/pharmacist/ambulances, clinical officer etc)?

Yes

No

Q9.Do you work with Ambulances/Emergency Medicine/Emergency Medical Service? (only asked if Yes to Q8, above)

Yes

No

Q10.What is your occupation? __________________________________________

**CPR Knowledge & Training**

Q11.Have you ever heard of CPR or cardiopulmonary resuscitation?

Yes

No

Q12.In your own words, what is CPR or cardiopulmonary resuscitation?

__________________________________________

Q13.Why would someone need CPR or cardiopulmonary resuscitation?

__________________________________________

Q14.Does CPR include chest compressions? (responses randomized)

Yes CPR includes chest compressions

No CPR does not include chest compressions

Not sure

Q15.Does CPR require "mouth to mouth"? (responses randomized)

Yes CPR requires "mouth to mouth"

No CPR does not require "mouth to mouth

Not sure

Q16.Have you ever taken a CPR or cardiopulmonary resuscitation training class?

Yes

No

Q17.When was the last time you attended a CPR or cardiopulmonary resuscitation training class? (only asked if Yes to Q16 above)

≤1 year ago

2 years ago

3 years ago

4 years ago

≥5 years ago

Q18.Would you be interested in receiving training in CPR or cardiopulmonary resuscitation? (responses randomized)

Yes

No

Not sure

**CPR Attitude**

Q19.I would give CPR or cardiopulmonary resuscitation to someone if they are... (Multiple response, responses randomized)

A child (mine or someone else's)

My spouse/partner (husband, wife, girlfriend,

boyfriend etc)

A stranger

My relative (brother, sister, in-law, nephew,

niece etc)

My neighbor

Other (specify)

I would not give anyone CPR

Q20.You are at home in the evening. Suddenly you hear a loud noise in the kitchen. You burst in and find a close family member lying lifeless on the floor. He or she is not breathing. You are alone in the house. What will you do? (Multiple response, responses randomized)

Call for ambulance

Call for taxi

Call for others to help

Give them mouth to mouth

Give them chest compressions

Do something else (specify)

I'm not sure what I will do

Q21.Why would you not want to give someone CPR or cardiopulmonary resuscitation? (Multiple response, responses randomized)

I fear I may catch a disease

I do not have the skills to give CPR

I may not know the person

(You can select more than one response) I do not have the confidence to give CPR

I may harm the person or make things worse

I do not want to give mouth to mouth

I am afraid of getting sued

Other (specify)

I will always give CPR
